# Self-Assembled Mucin-Containing Microcarriers via Hard Templating on CaCO_3_ Crystals

**DOI:** 10.3390/mi9060307

**Published:** 2018-06-19

**Authors:** Nadezhda G. Balabushevich, Ekaterina A. Sholina, Elena V. Mikhalchik, Lyubov Y. Filatova, Anna S. Vikulina, Dmitry Volodkin

**Affiliations:** 1 Department of Chemistry, Lomonosov Moscow State University, Leninskiye Gory 1-3, 119991 Moscow, Russia; nbalab2008@gmail.com (N.G.B.); sholina-katya@mail.ru (E.A.S.); luboff.filatova@gmail.com (L.Y.F.); 2 Federal Research and Clinical Centre of Physical-Chemical Medicine, Malaya Pirogovskaya, 1A, 119992 Moscow, Russia; lemik2007@yandex.ru; 3 Nottingham Trent University, School of Science and Technology, Clifton Lane, Nottingham NG11 8NS, UK; anna.vikulina@ntu.ac.uk

**Keywords:** CaCO_3_, mucin, adsorption, co-synthesis, layer-by-layer, protamine, aprotinin

## Abstract

Porous vaterite crystals of CaCO_3_ are extensively used for the fabrication of self-assembled polymer-based microparticles (capsules, beads, etc.) utilized for drug delivery and controlled release. The nature of the polymer used plays a crucial role and discovery of new perspective biopolymers is essential to assemble microparticles with desired characteristics, such as biocompatibility, drug loading efficiency/capacity, release rate, and stability. Glycoprotein mucin is tested here as a good candidate to assemble the microparticles because of high charge due to sialic acids, mucoadhesive properties, and a tendency to self-assemble, forming gels. Mucin loading into the crystals via co-synthesis is twice as effective as via adsorption into preformed crystals. Desialylated mucin has weaker binding to the crystals most probably due to electrostatic interactions between sialic acids and calcium ions on the crystal surface. Improved loading of low-molecular-weight inhibitor aprotinin into the mucin-containing crystals is demonstrated. Multilayer capsules (mucin/protamine)_3_ have been made by the layer-by-layer self-assembly. Interestingly, the deposition of single mucin layers (mucin/water)_3_ has also been proven, however, the capsules were unstable, most probably due to additional (to hydrogen bonding) electrostatic interactions in the case of the two polymers used. Finally, approaches to load biologically-active compounds (BACs) into the mucin-containing microparticles are discussed.

## 1. Introduction

The layer-by-layer (LbL) adsorption of oppositely-charged polymers (polyelectrolytes) onto matrices of various nature are actively used for the immobilization of biologically-active compounds (BACs) [1,2,3,4]. Bio-friendly loading of BACs into the vaterite CaCO_3_ crystals has been shown to be effective for encapsulation of fragile BACs into polymer-based microparticles assembled onto these sacrificial crystals [5,6,7]. The crystals coated by polymers can be eliminated in the presence of chelating agents, such as EDTA (ethylenediaminetetraacetic acid) or citric acid, or at pH below neutral.

The following synthetic biopolymers have been utilized for the preparation of biologically/medically-relevant microparticles: poly-L-lysine, poly-L-arginine, poly-L-glutamic acid, and poly-L-aspartic acid [8]. Natural biopolymers and their derivatives have also been used: sodium alginate, chitosan, pectin, gelatine, carrageenan, hyaluronic acid, chondroitin sulfate, dextran, and cellulose [8]. In general, natural polypeptides and polysaccharides are weak polyelectrolytes and can adopt multiple conformations in response to changes in the solution pH and temperature. The folding and unfolding of their chains is driven by a balance of a number of the internal interactions (hydrogen bonds, hydrophobic, electrostatic, etc.), providing fascinating properties for microparticles made of self-assembled natural biopolymers.

Nowadays one of the main research directions in microencapsulation through self-assembled nano- and micro-particles is devoted to looking for new biopolymers providing the microparticles with desired properties, such as biocompatibility, biodegradability, and mucoadhesiveness [9]. One of the major challenges is to efficiently load and adjust a sustained release of BACs that are typically weakly bound to polymer-based microparticles.

In the past decade an interest into mucins, mucoadhesive glycoproteins, has significantly increased [10,11,12,13,14,15]. Mucins are the main components of mucous membranes in the gastrointestinal tract, as well as nasal and oral mucous membranes. The mucins possess a function of a barrier for pathogens, as well as drugs.

**Figure 1:**
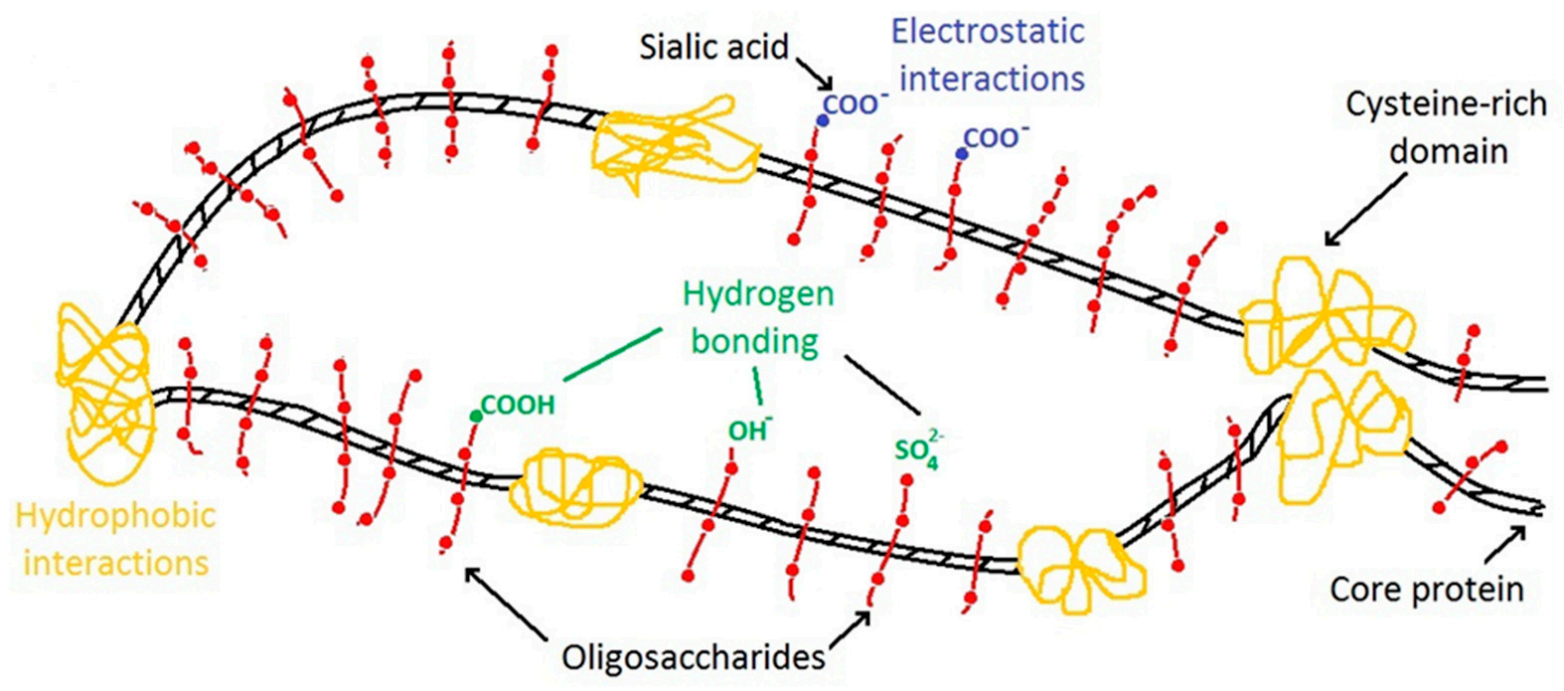
Schematic structure of mucin glycoproteins and their potentially mucoadhesive elements. More details can be found in [16].

Mucins are large, extracellular glycoproteins with molecular weights ranging from 0.5 to 20 MDa. Membrane-bound and secreted mucins are both highly glycosylated [10]. They consist of 80% carbohydrates: N-acetylgalactosamine, N-acetylglucosamine, fucose, galactose, and sialic acid (N-acetylneuraminic acid), and traces of mannose and sulfate. The oligosaccharide chains are attached to the hydroxyl side chains of serine and threonines in the protein core. They consist of 5–15 monomers, exhibiting moderate branching. The protein core itself is arranged into distinct regions. The central glycosylated region is comprised of multiple tandem repeats rich in serine, threonine, and proline (STP repeats). At the amino and carboxy terminals, as well as between STP repeats, there are regions of the second type with a small number of O-glycosylation and a few N-glycosylation sites. The content of cysteine in these regions is more than 10% and it participates in disulfide bond formation with subsequent dimerization and polymerization of the dimers to form multimers (Figure 1). Due to this structure a number of various interactions are present in mucins including electrostatics, hydrophobic interactions, and hydrogen bonding; these interactions largely define the properties of mucins [16].

Conformation of mucin is affected by pH and ionic strength [17]. Formation of self-assembled aggregates and gels is typical for mucins and is driven by the formation of S–S bonds and the interpenetration of the end glucose chains of mucin molecules [18] These processes, however, strongly depend on the source of mucin, purity, pH, and ionic strength [11,12,19].

Adsorption of mucin onto various solid surfaces has been reported, for instance onto hydrophilic and hydrophobic silica [20]. Elipsometry measurements have shown that adsorption is more pronounced at hydrophobic surfaces giving 1.2 and 3.8 mg of mucin per m^2^ of the hydrophilic and hydrophobic silica, respectively. Mucin can be strongly bound to 282 nm-sized nanoparticles of polystyrene resulting in 2.2 mg of mucin per one m^2^ of the nanoparticles [21]. After binding the nanoparticles were coated with 4–6 nm layer of mucin and possessed hydrophilic properties.

The negative charge of mucin at neutral and alkaline pH is due to the presence of sialic acids (pKa = 2.6) located on the ends of the chains of a polysaccharide backbone [11]. Mucins from various sources have been employed to assemble multilayers using the following polycations: polyallylamine hydrochloride, poly-L-lysine, polyethylenimine, methylcellulose, chitosan, and lactoperoxidases (Mw 78 kDa, pI 8.3) [20,21,22,23,24,25,26]. The thickness of the formed multilayers depends on pH and ionic strength.

Conformation of mucin molecules depends of the concentration of calcium ions [27,28,29,30]. Ca^2+^ ions have an effect on mucin aggregation, viscosity, and permeation of a mucous membrane for BACs and nanoparticles [28]. It is also known that Ca^2+^ has a significant influence on the formation of gallstones [27,31]. At the same time, to the best of our knowledge, there are no reports devoted to the interaction between mucin and the vaterite CaCO_3_ crystals.

This work aims at the development of new approaches for BAC microencapsulation based on the vaterite CaCO_3_ crystals and mucin. Here the mucin from the porcine stomach has been used for the naturally-derived mucin widely employed for research [11,25]. The porcine gastric mucin has been shown to be structurally related to human gastric mucin [32] and is, therefore, a decent substitute of human gastric mucin because of its high availability and reduced number of ethical issues required for the research. Moreover, mucins derived from a stomach have a tendency for gelation in acidic medium which may be important in order to form polymer-based microparticles when the CaCO_3_ crystals are eliminated in acidic medium. In order to test the applicability of mucin for the microencapsulation we focus on the following important aspects of the encapsulation process: (i) development of simple and robust analytical approaches (with no use of radiolabels and fluorescent probes) to determine native mucin in the presence of crystal and BACs; (ii) assessment of mucin loading into the crystals and multilayer capsules prepared based on mucin; and (iii) analysis of aprotinin (model BAC poorly loaded into the crystals [33,34]) encapsulation into the crystals in the presence of mucin.

## 2. Materials and Methods

Anhydrous calcium chloride, ≥93.0% (C1016), anhydrous sodium carbonate Na_2_CO_3_, ≥99.0% (S7795), commercial mucin from porcine stomach, Type III, (m1778), bound sialic acid 0.5–1.5%, protamine from salmon, fluorescein isothiocyanate isomer 1 (FITC), 2,4,6-trinitrobenzenesulfonic acid (TNBS), *N*-acetylneuraminic acid from *Escherichia coli*, ethylenediaminetetraacetic acid (EDTA), aprotinin, *N*-benzoyl-L-arginine ethyl ester (BAEE), gel filtration molecular weight markers kit MW 12–200 kDa (Sigma-Aldrich, St. Louis, MO, USA); trypsin from bovine pancreas (Fluka, Dresden, Germany); 5,5′-dithiobis(2-nitrobenzoic acid) (DTNB, Serva, Heidelberg, Germany); cysteine, >99.5% (BioUltra Sigma-Aldrich, St. Louis, MO, USA); Sephadex G-200 (Pharmacia, Stockholm, Sweden) were used. All chemicals for buffers were laboratory grade and purchased from Sigma–Aldrich (St. Louis, MO, USA). Before use, mucin solutions were sonicated for 30 min using the ultrasonic bath (Elmasonic S15H, Singen, Germany).

### 2.1. Analytical Determination of Mucin

The concentration of the glycoprotein mucin in solution was determined spectrophotometrically at the wavelengths of 214 nm and 260 nm, as well as by Sсhiff’s method via measurements of adsorption at 555 nm [35]. Analytical size exclusion chromatography in the Biofox 17 SEC 8 × 300 mm column (Bio-Works, Uppsala, Sweden) has been used utilizing the Smartline chromatographic system (Knauer, Berlin, Germany) in a solution of 0.15 М NaCl (Table S1). Preliminarily, the column was calibrated using solutions of purified mucin with different concentrations (0.01–0.1 mg mL^−1^) and proteins with different molecular weights (Figure S1, Table S1). A total of 0.2 mL of the mucin solution with a concentrations of 0.01–1.0 mg mL^−1^ were used for the chromatography analysis at the elution rate of 0.5 mL min^−1^. Absorbance of the eluted solutions was measured using the UV detector at wavelengths of 214 nm and 260 nm. After that the maximum absorbance of the high-molecular weight fraction was measured (the time of elution was 9.3–9.7 min).

### 2.2. Purification of Mucin via Chromatography

A total of 15–45 mL of the mucin solution (1–5 mg mL^−1^) was subjected to the column filled with Sephadex G-200 (dimension 2.5 × 35 cm) using the chromatographic system Bio-Logic LP (Bio-Rad, Hercules, CA, USA) in the solution of ammonia (pH of 9.0). The elution rate was 0.5 mL min^−1^, collection time for one fraction of eluted solution 12 min. Absorbance was determined in the obtained fractions at wavelengths of 214, 260, and 480 nm. The fractions containing mucin, as identified by absorbance and specific determination by the Schiff’s method (wavelength 555 nm), were combined and freeze-dried.

### 2.3. Synthesis of Mucin-FITC

Fifty milligrams of commercially available mucin was dissolved in 10 mL of 0.5 M carbonate buffer with pH 9.0. One millilitre of FITC solution (1 mg mL^−1^) in dimethylformamide was added drop by drop and the whole mixture was incubated for 24 h at 4 °C. The solution was chromatographed at the column filled with Sephadex G-200, as described above (Figure S2). The molar modification degree of the free amino groups in mucin-FITC was determined by titration with TNBS [36] and was found to be 17 ± 2% relative to purified mucin.

### 2.4. Synthesis of Desialylated Mucin

Fifteen milligrams of commercially available mucin was dissolved in 50 mL of 0.01 M HCl and incubated for 3 h at 80 °С. The solution was chromatographed at the column filled with Sephadex G-200, as described above (Figure S3). The content of sialic acids, determined by Hess’s method [37] using the calibration curve for N-acetylneuraminic acid, was found to be 2.30 ± 0.10, 1.80 ± 0.10, and 0.41 ± 0.05% for commercial, purified, and desialylated mucin, respectively.

### 2.5. Mucin Loading into the CaCO_3_ Crystals by Adsorption

The CaCO_3_ crystals were formed according to the standard procedure [5] by mixing of equimolar solutions of CaCl_2_ and Na_2_CO_3_. The formed crystals were washed twice with a pure water and dried at 70 °C. Thirty milligrams of the dry CaCO_3_ crystals were mixed with 1.5 mL of 1 mg mL^−1^ mucin solution. The suspension was agitated on a shaker for 30 min. The precipitate was separated by centrifugation (2 min, 1000× *g*) followed by removal of the supernatant and washed twice with 1.5 mL water. The content of mucin was analysed in the supernatant and the washing solutions.

Efficiency of mucin incorporation was calculated using the following equation:

$$\eta =\frac{\left(c_{0}-c_{e}\right)}{c_{0}}$$

where η is efficiency of protein incorporation, and *c*_0_ and *c_e_* are the initial and equilibrium protein concentrations, respectively (mg mL^−1^).

The amount of the loaded protein at equilibrium was calculated using the following equation:

$$q_{e}=\frac{\left(c_{0}-c_{e}\right)\cdot V}{m}$$

where *q_e_* is the adsorption capacity (mg g^−1^), *c*_0_ and *c_e_* are the initial and equilibrium protein concentrations, respectively (mg mL^−1^); *V* is the volume (mL) of the protein solution; and *m* is the mass (g) of CaCO_3_.

### 2.6. Mucin and Aprotinin Loading into the CaCO_3_ Crystals by Co-Synthesis

Three millilitres of the solution containing 1.67 mg mL^−1^ mucin and/or 0.167 mg mL^−1^ aprotinin were added to 1 mL of the solution 1 M CaCl_2_. The solution was stirred for 5 min (100 rpm), 1 mL of the solution of 1M Na_2_CO_3_ was added and further stirred for an additional 45 s. Then the suspension was incubated for 15 min. The precipitate was separated by centrifugation (2 min, 1000× *g*) and washed twice with 5 mL of water. If necessary, the particles were dried. The mass of the precipitate was determined, and the mass of CaCO_3_ was calculated assuming the complete process of crystallization of the insoluble crystals. The concentrations of mucin were determined in the supernatant and washing solutions and, if necessary, the activity of aprotinin was also determined. The concentration of active aprotinin in the solutions was determined by inhibition of trypsin using BAEE substrate as described in [34,38].

### 2.7. Preparation of Polyelectrolyte Microcapsules

The LbL deposition of either mucin (control experiment) or mucin and protamine (a pair of polymers used) has been performed on the synthetized CaCO_3_ crystals containing glycoprotein mucin preloaded by co-synthesis. In either case, the polymer concentration was 0.5 mg mL^−1^ and the crystals concentration was 20 mg mL^−1^. The coated crystals were washed twice with water and their ζ-potential was measured after each adsorption step. The CaCO_3_ matrix in the prepared particles coated with three mucin-protamine bilayers was dissolved by dropwise addition of an equimolar amount of 0.2 M EDTA (to solubilize all the crystals), and then the formed polyelectrolyte capsules were washed three times with water. The content of mucin in the microcapsules was determined by the analysis of its content in solutions obtained during the capsule preparation procedure.

### 2.8. Characterisation of the Crystals and Microcapsules

Analysis of microparticles prepared in this study was carried out using optical microscopy (Carl Zeiss, Jena, Germany), scanning electron microscopy (SEM, Zeiss DSM 40, Jena, Germany), and fluorescence microscopy (EVOS FL, Thermo Fisher Scientific, Waltham, MA, USA). Determination of the hydrodynamic diameter of commercial mucin (0.1 mg mL^−1^) was carried out using DLS (Malvern Zetasizer Nano ZS, Malvern, UK) and nanoparticle tracking analysis (NTA, Malvern NanoSight NS500, Malvern, UK). A suspension of microparticles (0.5 mg mL^−1^), and solutions of 0.1 mg mL^−1^ mucin or protamine were used for the analysis of the ζ-potential (ZP) using DLS.

## 3. Results and Discussion

### 3.1. Analysis of Mucin Purity via Permeation Gel Chromatography

The first step in this study was to develop an effective approach to determine mucin concentration in alkaline media (pH 7–10) and in the presence of BAC such as proteins and peptides. This is necessary because the CaCO_3_ crystals dispersed in water provide the alkaline pH due to hydrolysis of the CaCO_3_.

The classical way to determine mucin is based on Schiff’s method [35]. OH groups of mucin are oxidised by periodic acid to aldehydes (Figure S4). Our results indicate that pH of the analysed solution has a significant effect on the purple-violet colour of solutions (А_555_) obtained using this staining method (Figure S5). Thus, this method has been found as not useful for the determination of mucin concentration at alkaline pH.

**Figure 2:**
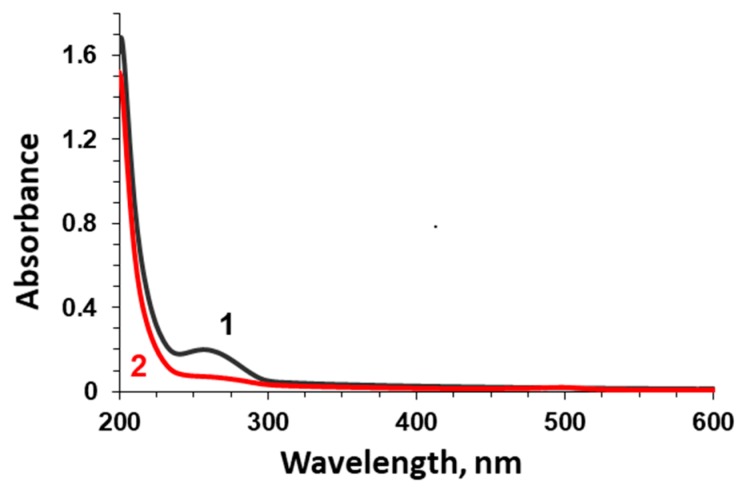
Absorption spectra of commercial (black line 1) and purified (red line 2) 0.1 mg mL^−1^ mucin.

A number of reports have identified some protein-based low-molecular-weight impurities in commercial samples of mucin [11,13,15,39]. Absorbance spectra of mucin used in this study show the presence of two well-defined peaks at 214 and 216 nm as shown in the Figure 2, line 1.

**Figure 3:**
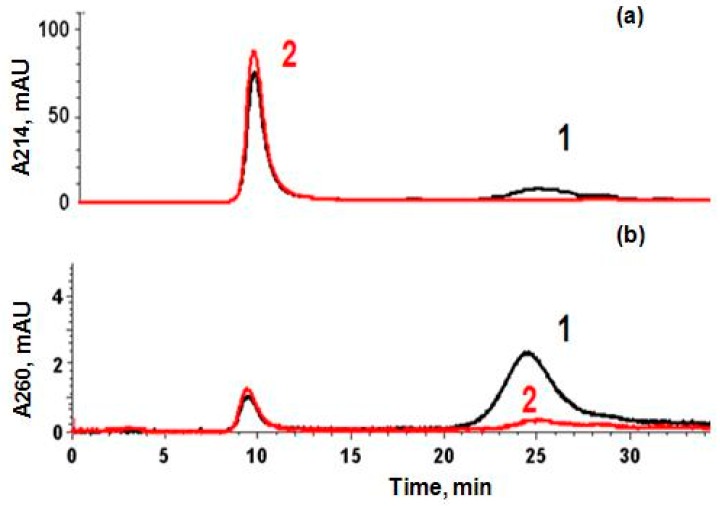
Elution profiles registered at 214 (**a**) and 260 nm (**b**) by analytical exclusion chromatography of commercial (black line 1) and purified (red line 2) mucins on a Biofox 17 SEC column (8 × 300 mm).

Analytical size exclusion chromatography has been employed for further analysis of commercial mucin revealing the presence of two fractions: low- and high-molecular-weight fractions (Figure 3, line 1). Only the high-molecular-weight fraction had absorption at both 214 and 260 nm and gave a specific staining by Schiff’s method. Another fraction (6–30 kDa) significantly contributed to the absorption at 260 nm is most probably the low-molecular-weight impurity of a protein nature.

**Figure 4:**
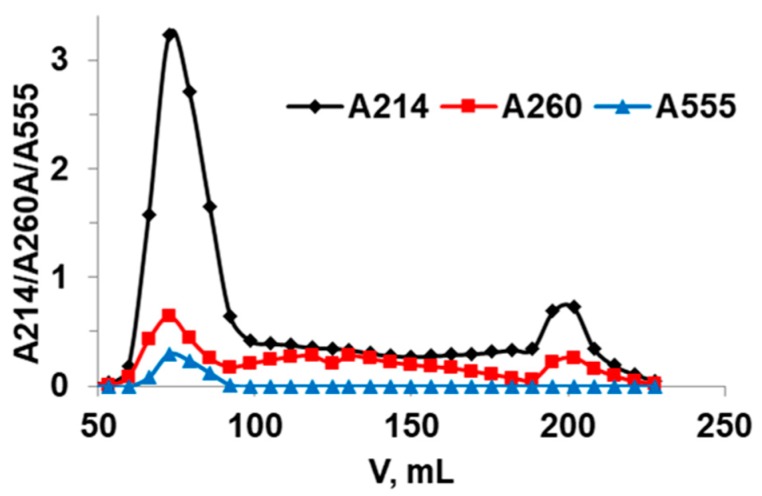
Elution profile obtained by gel filtration on Sephadex G-200 of commercial mucin.

To purify the commercial mucin, gel-permeation chromatography on Sephadex G-200 has been used (Figure 4). The high-molecular-weight fraction has been separated and lyophilized. This fraction has been further used in this study and is called purified mucin. Absorbance spectra (Figure 1) and chromatography profiles (Figure 3) of commercial and purified mucin confirm no protein-based impurities in the purified mucin. In addition, the purified mucin has been titrated by TNBS [36] showing a significant reduction of primary amino groups in purified mucin (38 per a mucin molecule) compared to the commercial one (164 per mucin molecule).

Further, the purified mucin has been used for the construction of calibration lines in order to determine glycoproteins in this study. Based on the results of gel-permeation chromatography, the content of glycoprotein in the commercial mucin has been found to be 87 ± 3% that is in line with results obtained using the Schiff’s method (content is 84 ± 2%).

### 3.2. Loading of Mucin into CaCO_3_ Crystals (Adsorption and Co-Synthesis)

**Figure 5:**
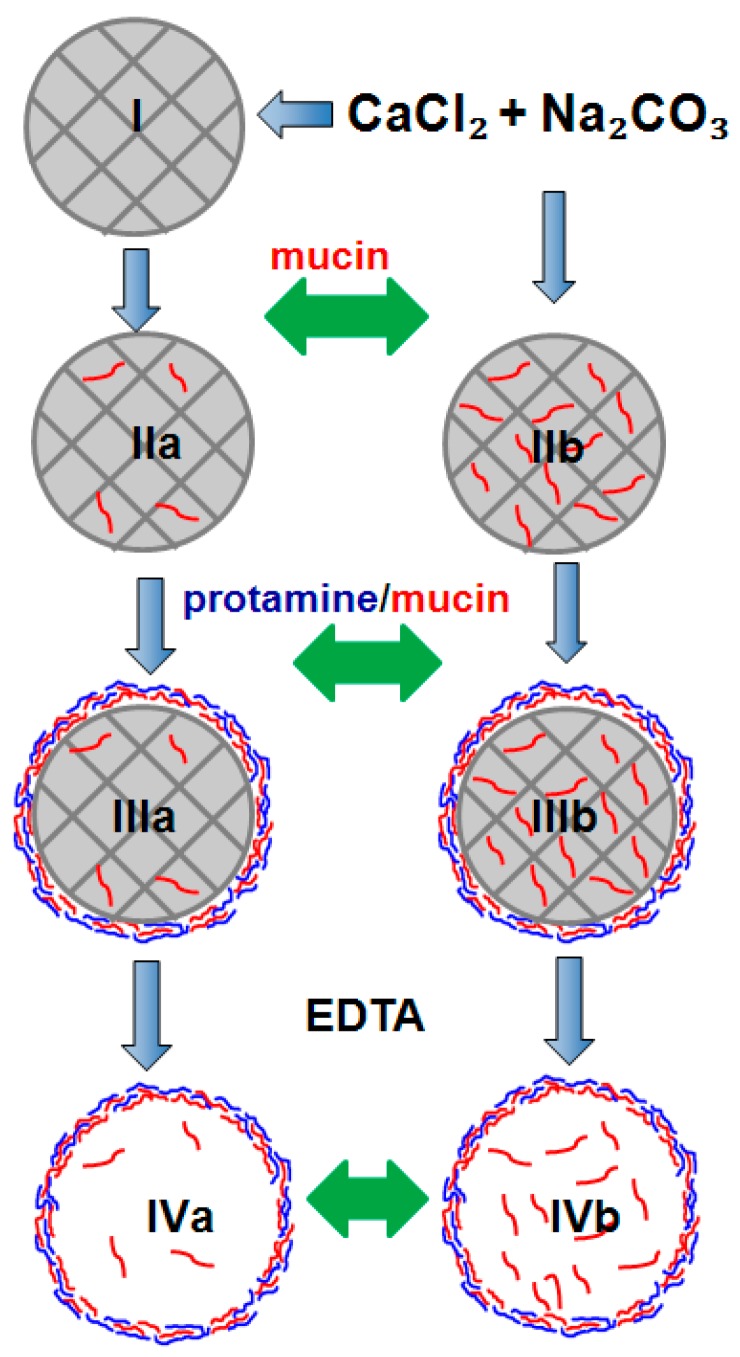
Schematics of formulation of microparticles (crystals and polymer capsules) using mucin: I–CaCO_3_ crystals; II–the crystals with mucin loaded by adsorption (**a**) or co-synthesis (**b**); III–crystals coated with three layer of oppositely charged protamine and mucin; IV–polymer-based microcapsules obtained after dissolution of the coated crystals using EDTA. Green arrows indicate a step of introduction of a BAC to be further encapsulated into the microparticles.

Loading of mucin into the crystals has been achieved via two methods, namely adsorption (mucin is adsorbed into performed crystals) or co-synthesis (mucin is trapped in the crystals during crystal synthesis). Figure 5 shows the schematics of the approaches used. Of note, the conditions of mucin loading have been kept identical for both loading methods, such as the same time of exposure of mucin to the crystals (15 min), the same final crystal and mucin concentrations (20 and 1 mg mL^−1^, respectively), etc. This gives an option to compare the methods.

**Figure 6:**
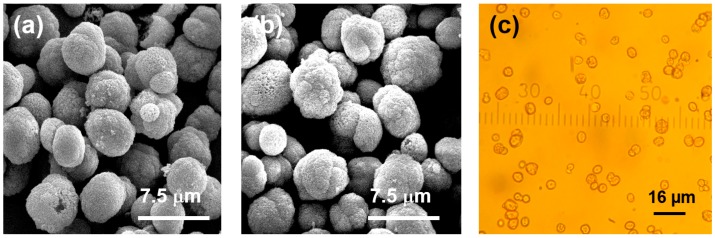
SEM (**a**,**b**) and optical (**c**) microscopy images of CaCO_3_ crystals with adsorbed (**a**) and co-synthetized mucin (**b**,**c**).

Optical and SEM microscopies did not reveal significant differences in morphology and size (3–5 µm) of the crystals before and after mucin loading (Figure 6). This may be related to the rather low concentration of mucin used. We believe that analysis of the surface of the crystals is sufficient to conclude about the crystal internal structure change. This is because we have found (by atomic force microscopy (AFM) measurements and other relevant techniques) that there is direct correlation between the size of nanocrystallines on the surface of the crystals and those in the internal volume [40]. It would be of interest to analyse the morphology of the crystals using AFM in the future in order to get more insights into the internal structure of the crystals. In this work we use optical microscopy and SEM images for identification of the polymorph form of the crystals since the vaterite form has a typical spherical shape, compared to cubic calcite. The same crystals, as obtained in this study, have been previously analysed by X-ray diffraction analysis (XRD) revealing the vaterite form [40]. In the future we plan to utilize XRD as a powerful tool to analyse the structure of the crystals loaded with substantial amounts of mucin under various conditions. This will open a way to probe the effect of the preparation conditions on the crystal structure.

Loading efficiency for mucin loading into the crystals and the amount of mucin released from the crystals during washing with water have been worked out by analysis of supernatants of solutions obtained during the mucin loading procedure (Table 1). Loading by co-synthesis resulted in almost twice higher content of mucin in the crystals. Moreover, mucin loaded by co-synthesis has, to a higher extent, been retained in the crystals during crystal washing with water. These results corroborate well with findings reported for proteins [33] showing the same trend. Based on the surface area of CaCO_3_ crystals obtained using the same procedure as in this study (8.8 m^2^ g^−1^ [41]) the mucin adsorption per a unit surface area of the crystals can be calculated. This value has been found to be 1.25 mg m^−2^, which is similar to mucin adsorption onto SiO_2_, which gives 1.2 mg m^−2^ [21]. Better understanding of the effect of the hydrophobicity of the surface of the crystals and limitations for mucin diffusion through the pores of the crystals onto the loading amount can be realised in our future work.

Further, we have analysed an average hydrodynamics diameter of mucin in water solution (1 mg mL^−1^). DLS has revealed two populations of molecules of sizes 40 and 250 nm, respectively. This is in a line with results obtained for commercial mucin from other sources [19]. The fraction with larger size was diminished as a result of ultrasound treatment, dilution of mucin solution, increase of pH (Figure S6). Both fractions are characterized by a negative value of the zeta potential (−15 mV). We believe that the fraction with the larger size (250 nm) corresponds to the aggregated mucin and the smaller fraction (40 nm) belongs most probably to single mucin molecules.

**Figure 7:**
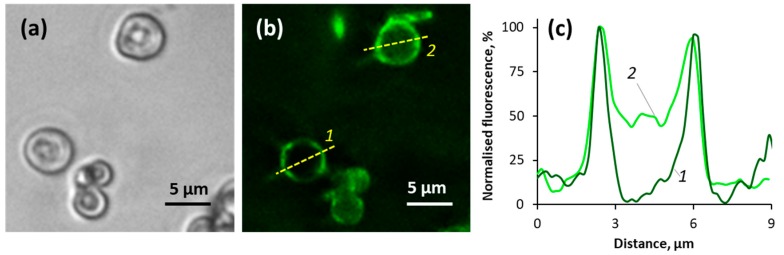
Transmittance (**a**) and fluorescence (**b**) microscopy images of vaterite crystals (4 mg mL^−1^ CaCO_3_) after incubation with mucin-FITC (1 mg mL^−1^) for 15 min; and (**c**)-fluorescence profiles taken along the yellow lines 1 and 2 across the particles in the image (**b**).

Literature reports indicate that intermolecular interaction between mucin molecules can be enhanced in the presence of Ca^2+^ [28] and hydrodynamic radius of mucin in the solution of CaCl_2_ is reduced compared to that in water [29]. We hypothesize that the smaller fraction of mucin molecules (single mucin molecules) can diffuse through pores of the crystals (pore size in the range 5–40 nm [42] and the larger fraction would be located presumably on the crystal surface. This may be valid for both methods of mucin loading into the crystal, i.e., adsorption and co-synthesis. In order to prove this assumption, we have analysed the mucin distribution in the crystals using fluorescence microscopy and mucin-FITC (Figure 7). The results demonstrate that mucin is predominately located on edges of the crystals and partially penetrates inside the crystal pores, which supports the assumption above. In the previous work we have found that 70 kDa poly(sodium 4-styrenesulfonate) and poly(allylamine hydrochloride) do not permeate well through pores of the crystals made at 22 °C (same protocol as for the crystals prepared in this study) [40]. These polymers are supposed to have smaller size than 40 nm (the small fraction of mucin identified as mentioned above). However, multiple adsorption of these synthetic polymers by the LbL manner and their high charge may limit their diffusion into the crystals.

Loading of desialylated mucin into the CaCO_3_ crystals has been investigated in order to probe an effect of sialic acids. The loading efficiency of the desialylated mucin was found to be 33 and 36% lower compared to that for the loading of commercial mucin using adsorption and co-synthesis, respectively. At the same time, the loss of mucin during washing in water increased by 10–15% for either loading approaches. Thus, we can conclude that the presence of sialic acids in mucin most probably improves the loading and retention of mucin in crystals, which can be explained by the interaction of the acids with Ca^2+^ provided from the crystals. Binding of desialylated mucin with the crystals can be driven by the interaction of Ca^2+^ with the protein-based part of mucin [29].

### 3.3. Encapsulation of Aprotinin into Mucin-Containing CaCO_3_ Crystals

Low-molecular-weight protein aprotinin (MW 6.5 kDa, pI 10.5) is an inhibitor of proteolytic enzymes and is actively used as a medicine [43]. It has recently been shown [33,34] that positively-charged aprotinin does not lose its biological activity in the presence of the CaCO_3_ crystals. However, its loading into the crystals by adsorption or co-synthesis is rather low compared to other proteins, such as insulin and catalase; moreover, the retention of aprotinin in the crystals is not high in washing steps with water.

We have further tested whether aprotinin loading into the crystals can be improved via co-loading of mucin into the crystals. Co-loading of mucin resulted in an increase of aprotinin content by a factor of three, giving a high content of aprotinin in the crystals of 1.5 ± 0.2 mg g^−1^ after two washing steps with water. We believe that the formation of inter-polyelectrolyte complex between mucin and aprotinin is a reason of better retention of aprotinin in the formed hybrid crystals.

### 3.4. Mucin-Containing Polymer Multilayer Capsules

**Figure 8:**
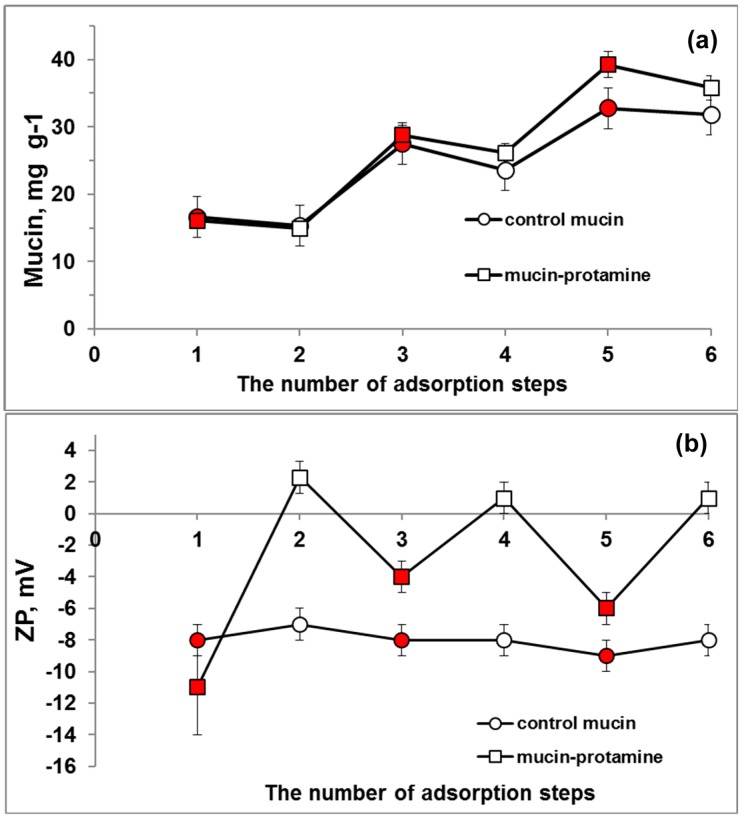
The content of mucin (**a**) and the zeta potential (**b**) of mucin-containing crystals (*n* = 1) as a function of a number of deposition steps of either mucin or water (control) or mucin and protamine pairs. Тhe adsorption of mucin is indicated with a red colour.

In this part of the work we have considered an option to utilize mucin for the formulation of multilayer capsules made of mucin and protamine as oppositely-charged bio-polymers. For this, mucin-containing crystals were coated by mucin and protamine layers in the LbL manner. The peptide protamine (5 kDа, pI 10.5) [44] has been previously used as a polycation [34,45] and, similar to aprotinin, it did not show high affinity to the CaCO_3_ crystals [33]. The zeta potential of protamine in water has been found to be +(7 ± 3) mV. During the LbL polymer deposition onto the crystal, the Zeta potential of the coated crystals has been reversed from negative values (mucin deposition) to positive ones (protamine deposition), as shown in Figure 8b. This proves the formation of the mucin-protamine complex upon the coating procedure. Protamine has absorption maxima at 214 nm and this is why the inclusion of mucin into the crystals has been determined using gel-permeation chromatography. Protamine adsorption resulted in the removal of a part of previously-deposited mucin (Figure 8a). However, the trend of the bio-polymer deposition demonstrated an increase of a total amount of the adsorbed polymers as a whole. The deposition process is most probably driven by the formation of both electrostatic interactions between the polymers and hydrogen bonding as well. The similar deposition behaviour has been reported for bovine submaxillary mucin in combination with polyallylamine hydrochloride [23] or lactoperoxidase [20].

Deposition of one polymer, namely mucin, has been used as a control experiment (Figure 8). In this case, the crystals have been incubated stepwise in mucin solution and in water. The deposited sequence can be shown as СаСО_3_(mucin)_3_ since the incubation in water is typically used as an intermediate step between the deposition of polymers to wash out weakly-adsorbed polymer molecules. The zeta potential of the coated particles has not been changed with an increase of the number of deposited layers of mucin (Figure 8b). At the same time, the total amount of adsorbed mucin has been increased with each deposition layer (Figure 8a). This is most probably due to adsorption of more than one layer of mucin. The Ellman method [46] did not reveal any free SH-groups in the commercial mucin sample, meaning that disulphide bonds cannot be responsible for intermolecular interactions between the mucin molecules. It is of note that an amount of adsorbed mucin in the controlled experiment was slightly lower than that for the mucin-protamine coating (Figure 8a). This stimulated us to hypothesize that the mucin-protamine interactions are driven by both electrostatics and hydrogen bonding (Figure 1) and the sequential deposition of only mucin (control experiment) takes place due to only hydrogen bonding allowing the deposition of multiple layers of only mucin. In future research, in order to probe the hydrogen bonding, we plan to prepare the crystals with higher mucin content and utilize Fourier-transform infrared (FTIR) spectroscopy as a convenient way of analysing mucin [47]. In this work, the rather low loading concentration of mucin limits us for FTIR study.

**Figure 9:**
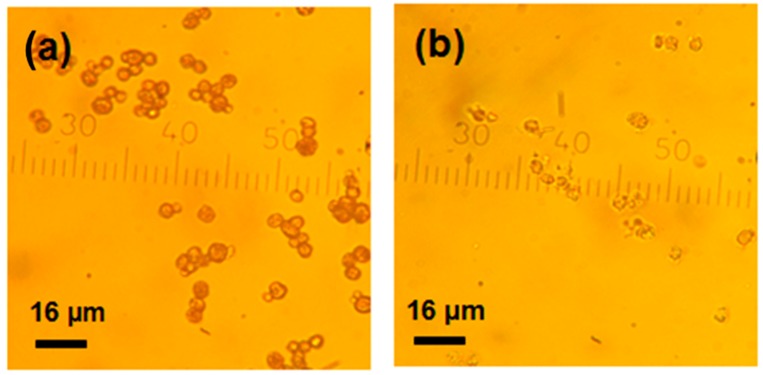
Optical microscopy images of polymer-coated crystals СаСО_3_(mucin-protamine)_3_ (**a**) and multilayer capsules (mucin-ptotamine)_3_ formed after addition of EDTA to the coated crystals (**b**). The CaCO_3_ crystals contain mucin loaded by co-synthesis.

The coated crystals СаСО_3_(mucin-protamine)_3_ and СаСО_3_(mucin)_3_ crystals-were stable upon storage in water at 4 °C for a month without any sign of recrystallization (Figure 9 and Figure S7). The presence of 0.2M EDTA resulted in dissolution of the carbonate crystals followed by formation of stable (mucin-protamine)_3_ capsules (Figure 9b). At the same time, stable capsules solely made of mucin have not been formed. This confirms that electrostatic interactions play a crucial role in the formation of stable multilayers and may be the contribution of electrostatics is stronger than that of hydrogen bonds, stressing the dominating role of electrostatics.

### 3.5. BAC Loading into Mucin-Containing Microparticles

Based on the results obtained using proteins (protamine, aprotinin), one can expect that the mucin-containing particles may be effective for loading of BAC, in particular small-molecular-weight and positively charged BACs that are difficult to load in substantial amounts. A rationality for the loading of such BACs should be driven by the nature and a charge of the components (Figure 2, indicated by green arrows). One can expect three main methods to load BACs: (i) via adsorption or co-synthesis into the crystals together with mucin; (ii) as polycations during sequential deposition of multilayers; and (iii) post-loading into preformed mucin-containing polymer capsules. The presence of mucin in the CaCO_3_ crystals or multilayer capsules makes the crystals and capsules extremely attractive for mucoadhesive delivery of BACs through a mucous membrane [15,48,49,50]. In regards to this, the LbL assembly will provide a strong tool for introduction of various components into the assembled structures in order to tune a function of the finally-assembled structure. Novel approaches to probe diffusion into the formed multilayers are essential to understand and control a structure of the assemblies and release characteristics of BACs from the assembled structures [51,52].

We believe that the approaches demonstrated here for assembly of mucin-containing polymer-based microparticles can be further used for the design of composite multifunctional micro- and nano-particles with required applications. For instance, introduction of the protein-repelling agent polyethylene glycol into the particles by the approaches developed earlier [53,54] may reduce protein-binding to the particles if undesired. Loading of a thermo-sensitive polymer, such as poly(N-isopropilacrylamide), can give an option to assemble particles able to change their size and hydrophobicity upon the temperature increase at physiologically-relevant conditions [55]. Utilization of mucin-containing CaCO_3_ crystals for fabrication of porous self-assembled alginate hydrogels [56,57,58] may be beneficial to design the hydrogels for the engineering of tissue having contact with mucosa. New perspectives are open to assemble mucin-containing particles of various shapes via hard templating onto protein aggregates as demonstrated earlier [59,60,61,62]. This approach may further be considered in our upcoming study.

## 4. Conclusions

This study demonstrated the development of novel approaches for encapsulation of mucin into self-assembled microstructures, i.e., polymer-based microparticles templated onto vaterite CaCO_3_ crystals. The following analytical methods for determination of mucin in alkaline solutions and in the presence of the crystals and proteins have been adopted: spectrophotometrical analysis of adsorption at 214 nm and analytical permeation chromatography using a Biofox 17 SEC column. The analytical methods used demonstrated the presence of protein-based low-molecular-weight fraction in commercial mucin samples that had been removed to obtain purified mucin for further use.

Loading of mucin into the crystals by co-synthesis has been shown to be more effective than the loading by adsorption (the loading capacity 11 and 6 mg of mucin per gram of the crystals, respectively). Mucin can aggregate in water solution giving aggregates with a hydrodynamic diameter of a few hundreds of nm, the diameter of single mucin molecules (in equilibrium with the aggregates) is around 40 nm. As proven by optical fluorescence imaging and DLS mucin molecules can adsorb onto and diffuse into the porous crystals that have pore size of the same dimensions (pores are in the range 20–60 nm). Most probably the bulky and highly hydrated mucins are able to squeeze out through the pores. Interestingly, desialylated mucin demonstrated weaker binding to the crystals that can be explained by an enhancement of the binding via the interaction of calcium ions with sialic acids present in the mucin backbone. Moreover, the presence of the sialic acids improved retention of mucin in the crystals upon water washings.

Loading of mucin into performed CaCO_3_ crystals can be achieved via LbL deposition of a single mucin or mucin-protamine pair, as proven by monitoring of the zeta potential and the amount of deposited compounds. Stable multilayer capsules can, however, be formed after crystal elimination by EDTA only for mucin-protamine pairs. This can be explained by additional electrostatic interactions for the pair compared to only hydrogen bonding in the case of mucin deposition as a single component.

Successful inclusion of BACs into mucin-containing microparticles is demonstrated for aprotinin as an important protease inhibitor. Finally, approaches for BAC loading are discussed in regards to the encapsulation strategy proposed based on the CaCO_3_ crystal. The results of this study may open new perspectives to utilize mucin as an important mucoadhesive polymer for effective encapsulation of various compounds, including BACs.

## Figures and Tables

**Table 1:** Characteristics of the incorporation of commercial mucin (1 mg mL^−1^) into the CaCO_3_ crystals (20 mg mL^−1^).

Loading Method	Efficiency of Mucin Loading, %		Release after Washing, % of Loaded	ZP, mV
	Spectrophotometry	Analytical Chromatography		
Adsorption	12 ± 2	10 ± 1	11 ± 1	−(15 ± 3)
Co-synthesis	22 ± 3	18 ± 2	5 ± 1	−(11 ± 2)

## Supplementary Materials

The following are available online at http://www.mdpi.com/2072-666X/9/6/307/s1, Figure S1: Calibration curve used for the determination of the concentration of mucin by analytical exclusion chromatography using Biofox 17 SEC in 0.15 M NaCl solution by measurement of absorbance of eluted samples at 214 nm with a release time of 9.3–9.7 min; Figure S2: Gel-permeation chromatography of mucin-FITC using Sephadex G-200; Figure S3: Gel-permeation chromatography of desialylated mucin using Sephadex G-200; Figure S4: Scheme of quantitative determination of mucin by the Schiff method; Figure S5. Influence of the tested media on the determination of mucin by the Schiff method; Figure S6. Typical hydrodynamic diameter distribution for commercial mucin (1 mg mL^−1^, H_2_O, 250 °C); Figure S7. Optical microscopy images of vaterite crystals before (a) and (b) after coating with (mucin)3; Table S1. Calibration curves used in the work.
