# Supplementary material for: Self-Assembled Mucin-Containing Microcarriers via Hard Templating on CaCO3 Crystals
Source: Micromachines (Basel). 2018 Jun 19;9(6):307. doi: 10.3390/mi9060307 (PMC6187553; doi:10.3390/mi9060307)
Supplement: Supplementary file 1 [file micromachines-09-00307-s001.pdf]

Supplementary Materials

Self-Assembled Mucin-Containing Microcarriers via Hard Templating on $CaCO_3$ Crystals

Balabushevich G. Nadezhda[1], Sholina A. Ekaterina[1], Mikhalchik V. Elena[2], Filatova Y. Lyubov[1], Vikulina S. Anna[3], Volodkin Dmitry[1,3]*

[1] Lomonosov Moscow State University, Department of Chemistry, Leninskiye Gory 1-3, 119991 Moscow, Russia, nbalab2008@gmail.com, sholina-katya@mail.ru, luboff.filatova@gmail.com
[2] Federal Research and Clinical Centre of Physical-Chemical Medicine, Malaya Pirogovskaya, 1A, 119992 Moscow, Russia, lemik2007@yandex.ru
[3] Nottingham Trent University, School of Science and Technology, Clifton Lane, NG11 8NS Nottingham, UK, anna.vikulina@ntu.ac.uk, dmitry.volodkin@ntu.ac.uk
* Correspondence: dmitry.volodkin@ntu.ac.uk; Tel.: +44-115-848-3140

Table S1. Calibration curves used in the work.

Method	The Equation	R^2
Schiff	$A_{555} = 8.8561x$	0.9955
Spectrophotometric	$A_{214} = 7.2304x$	0.9996
	$A_{216} = 2.5399x$	0.9993
Analitical chromatography on Biofox 17 SEC: 1) Determination of mucin concentration by the A_{214} fraction with a release time of 9.3–9.7 min. 2) Determination of molecular weight	$A_{214} = 0.838x$<br>log Mw = –0,1072t + 6,6768, where t is the time of the fraction exit from the column, min	0.9970<br>0.9648

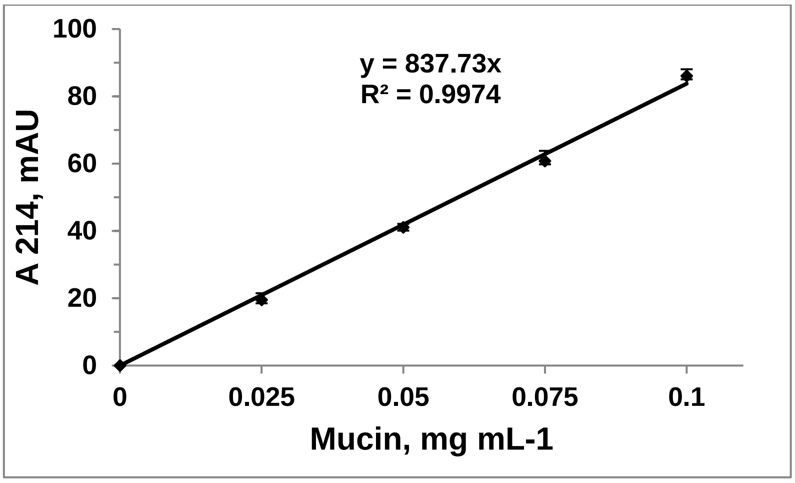

Figure S1. Calibration curve used for the determination of the concentration of mucin by analytical exclusion chromatography using Biofox 17 SEC in 0.15 M NaCl solution by measurement of absorbance of eluted samples at 214 nm with a release time of 9.3–9.7 min.

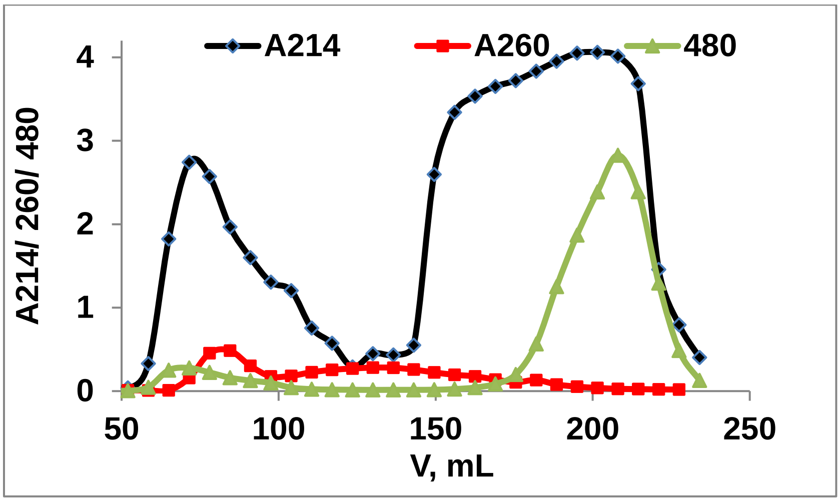

Figure S2. Gel-permeation chromatography of mucin-FITC using Sephadex G-200. The individual eluted fractions have been taken and the absorbance of the samples has been measured at 214, 216, an 480 nm.

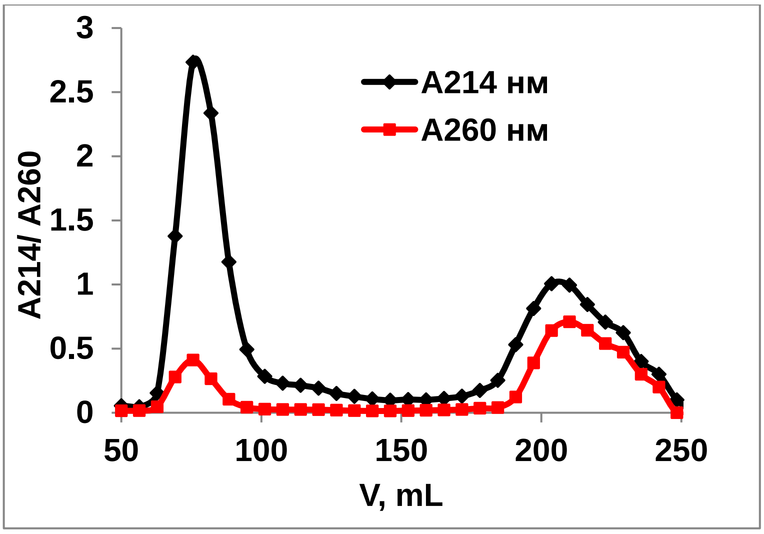

Figure S3. Gel-permeation chromatography of desialated mucin using Sephadex G-200. The individual eluted fractions have been taken and the absorbance of the samples has been measured at 214 and 216 nm.

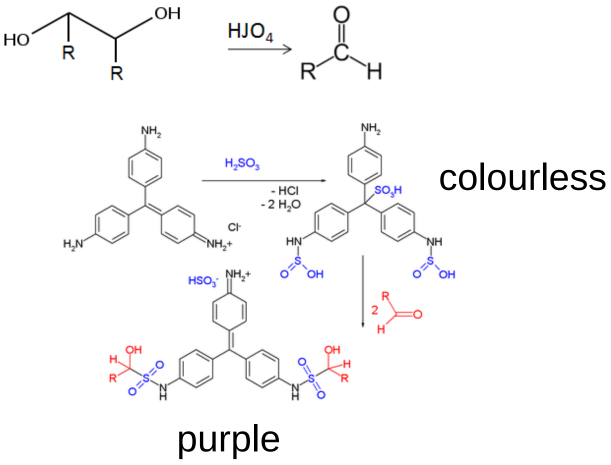

Figure S4. Scheme of quantitative determination of mucin by the Schiff method.

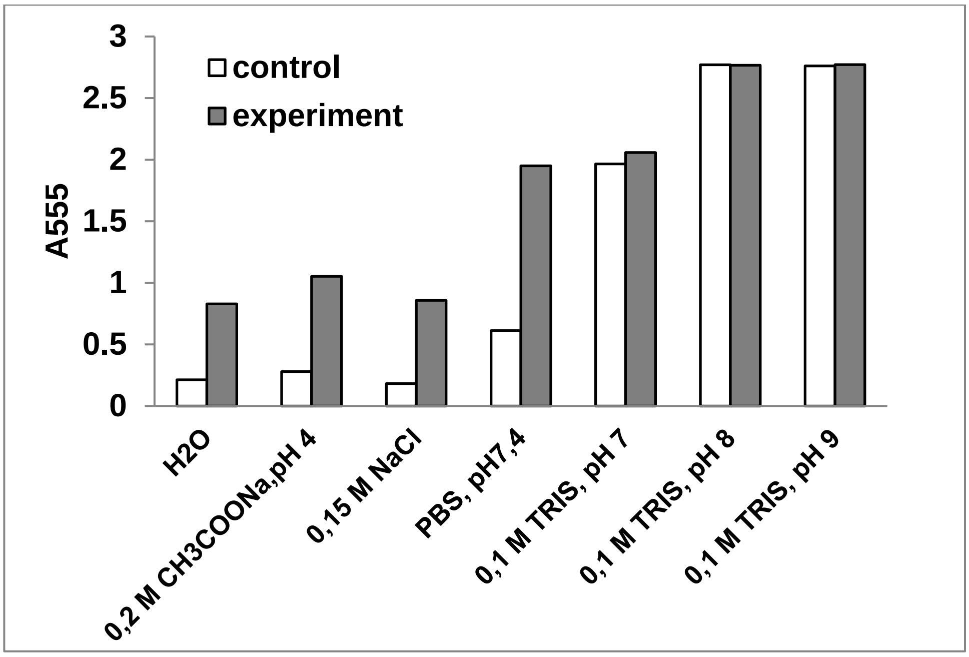

Figure S5. Influence of the tested media on the determination of mucin by the Schiff method. The control sample did not contain mucin; the tested samples contained 0.1 mg mL^{-1} mucin.

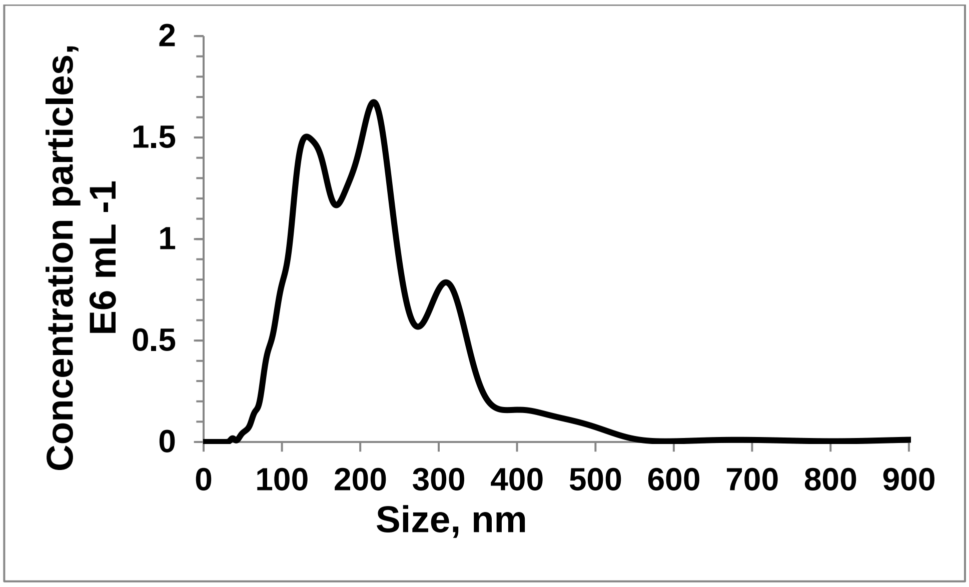

Figure S6. Typical hydrodynamic diameter distribution for commercial mucin (1 mg mL^{-1}, H_2O, 25 °C) samples as measured by NTA.

RESULTS:
Size distribution: mean: 238 nm, mode: 217 nm, SD: 157 nm
Cumulative data (nm): D10: 115, D50: 208, D90: 354, D70: 253
User lines: 0 nm, 0 nm
Total concentration: 21.84 particles/frame, 3.13 × 10^{-8} particles/mL
Selected concentration: 0.00 particles/frame, 0.00 × 10^{-8} particles/mL
Fitted curve : mean: 0 nm, SD: 0
Completed tracks: 529
Drift velocity: 1300 nm/s

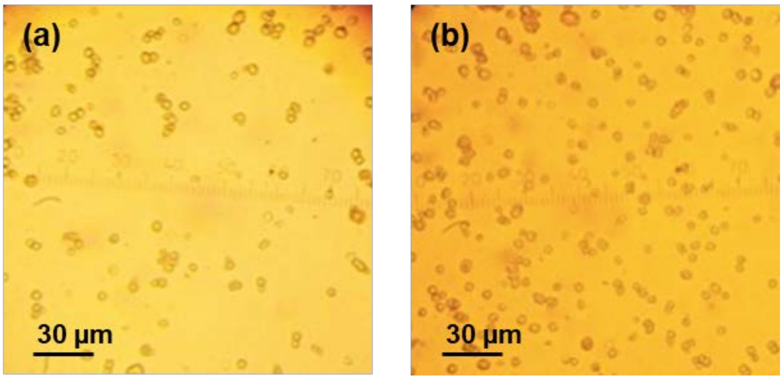

Figure S7. Optical microscopy images of vaterite crystals before (a) and (b) after coating with $(mucin)_3$. The crystals contain co-synthesised mucin. Magnification x40.
